# The Impact of Cladribine Tablets on Cognition Over One Year in Patients With Multiple Sclerosis: A Preliminary Study

**DOI:** 10.1002/brb3.71844

**Published:** 2026-10-28

**Authors:** Alexis M. Callen, Bonnie I. Glanz, Shahamat Tauhid, Brian C. Healy, Ariele Jinich, Samar Khalil, Maria K. Houtchens, Farrah J. Mateen, Salvatore Napoli, Syed Rizvi, Tarun Singhal, Jonathan Zurawski, Rohit Bakshi

**Affiliations:** ^1^ Brigham MS Center Department of Neurology Brigham and Women's Hospital/Mass General Brigham, Harvard Medical School Boston Massachusetts USA; ^2^ Department of Neurology Massachusetts General Hospital, Harvard Medical School Boston Massachusetts USA; ^3^ Neurology Center of New England Foxborough Massachusetts USA; ^4^ Multiple Sclerosis Center of Rhode Island Warren Alpert Medical School of Brown University Providence Rhode Island USA

**Keywords:** brain, cladribine, cognition, cognitive testing, multiple sclerosis, treatment

## Abstract

**Introduction:**

Cognitive impairment is common in multiple sclerosis (MS), affecting >40% of patients. Cladribine tablets are a high‐efficacy MS disease‐modifying therapy to prevent relapses and limit disease progression. Our previous study showed cortical gray matter lesion stability over one year after the start of cladribine, though its effect on cognition is not well known. This study aimed to investigate the one‐year impact of cladribine tablets on cognition in multiple sclerosis.

**Methods:**

Fourteen MS subjects [age (mean ± SD) 50.2 ± 10.8, 7.14% men, 57.1% relapsing–remitting MS (*n = *8), 42.9% secondary progressive (*n = *6), expanded disability status scale 4.1 ± 2.1] were administered the Brief Repeatable Battery of Neuropsychological Tests (BRB‐N) and the Stroop Color and Word Test (Stroop) at an average of 1.5 months from cladribine start. The BRB‐N measures information processing speed (Symbol Digit Modalities Test, Paced Auditory Serial Addition Test [PASAT]), verbal memory (Selective Reminding Test [SRT]), visual‐spatial memory (10/36 Spatial Recall Test), and verbal fluency (Word List Generation). Stroop assesses processing speed and executive function. When available, alternate versions of the individual cognitive tests were used at follow‐up to reduce practice effects. Paired *t*‐tests compared within‐subject baseline and follow‐up data.

**Results:**

After one year, no worsening was noted on any cognitive test. Furthermore, both PASAT 3‐second total correct and SRT total delayed recall trended toward improved scores on‐study, with an average 3.4 (*p = *0.07) and 1.1 (*p = *0.05) point increase, respectively.

**Conclusion:**

In this preliminary study, no clear decline in cognitive performance was detectable over one year in patients newly started on cladribine tablets.

## Graphical abstract

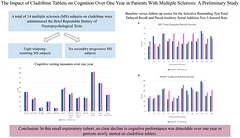

## 1 Introduction

Cognitive impairment affects more than 40% of patients with multiple sclerosis (MS) (Rao et al. 1991) and often worsens over time (Strober et al. 2014; Amato et al. 2001), with deficits present even in early disease stages. (Glanz et al. 2007; DiGiuseppe et al. 2018) The most common cognitive deficit in MS is reduced information processing speed, which is associated with employment status, health‐related quality of life, and social engagement (MacAdam et al. 2023; Glanz et al. 2010; Honarmand et al. 2011). Mitigating these changes is vital for patient autonomy and quality of life.

Cognitive impairment is associated with various MRI measures of tissue destruction such as whole brain (Houtchens et al. 2007), cortical gray matter (GM) (Eijlers et al. 2018; Damjanovic et al. 2017), hippocampal, and deep GM atrophy (Houtchens et al. 2007; Damjanovic et al. 2017). Measures of active and chronic inflammation are also associated with cognition. Preziosa and colleagues compared groups of MS patients with cognitive impairment versus preservation and found that the number of patients with at least one paramagnetic rim lesion was significantly higher in the impaired group (Preziosa et al. 2024). T1 hypointense and T2 white matter lesions and cortical lesions are also associated with cognitive impairment (Houtchens et al. 2007; Damjanovic et al. 2017; Mike et al. 2011). These measures offer potential targets for disease‐modifying therapies (DMTs) which could in turn limit cognitive symptoms. The effect of DMTs on cognition is an area of continued research in MS, but the impact of newer therapies, such as oral cladribine, is not well known.

Cladribine tablets are an FDA‐approved DMT that target lymphocytes, reducing T and B cell activity (Giovannoni et al. 2018). Clinical trials and real‐world studies have shown that cladribine limits clinical relapses and reduces the risk of sustained progression of physical disability in MS (Giovannoni et al. 2018; Aerts et al. 2023; Giovannoni et al. 2023). Studies have also shown that cladribine limits MRI activity as assessed by gadolinium‐enhanced and T2 lesion counts (Aerts et al. 2023; de Stefano et al. 2022; Freedman et al. 2021; Comi et al. 2018). Finally, cladribine is linked to limiting MRI‐defined damage to the GM, including cortical GM lesions (Zurawski et al. 2025), global GM volume (Cortese et al. 2023), and deep GM volume (Barrantes‐Cepas et al. 2025).

Since cladribine tablets impact MRI findings related to cognitive impairment (white matter lesions and GM damage), this therapy could play a role in limiting cognitive decline. Previous studies assessing cognitive performance of MS patients on cladribine support this idea. Glasmacher et al. (2022) assessed “hidden” disability (depression, pain, anxiety, fatigue, sleep difficulties, and cognitive decline), with cognitive decline based on the Paced Auditory Serial Addition Test (PASAT), over 12 months in MS patients on various DMTs. They found that patients treated with cladribine or fingolimod showed improvement on various measures including cognitive measures. Furthermore, Conway et al. (2025) found that processing speed was stable over time in cladribine‐treated patients measured in six‐month intervals over two years.

The goal of our study was to test the effects of newly started cladribine on a range of cognitive domains over one year early in the start of therapy to provide further understanding and validation of its role in MS treatment.

## 2 Materials and Methods

### 2.1 Subjects

Cohort characteristics at baseline and follow‐up are shown and compared in Table 1. A total of 14 MS patients (8 relapsing–remitting and 6 secondary‐progressive) who recently began or planned to start their first dose of cladribine tablets participated in the study. All patients were administered cladribine following prior exposure to other DMTs, as shown in Table 2. Subject inclusion criteria were: 1) ages 18–65 years, 2) no major medical comorbidities, and 3) no clinical relapses or corticosteroids one month prior. Thirteen patients had Expanded Disability Status Scale (EDSS) ratings at baseline and thirteen at follow‐up, by an MS neurologist on the day of cognitive testing. These patients were part of a separate report on 7T MRI and physical disability aspects of cladribine therapy (Zurawski et al. 2025).

**TABLE 1:** Cohort characteristics.

	Baseline	∼1 year follow‐up	*p*‐value
Age (years)	50.2 ± 10.8 [34.8–65.1]	51.3 ± 10.9 [35.8–66.2]	—
Women, *n* (%)	13 (92.9%)	13 (92.9%)	—
Disease duration (years)^a^	12.7 ± 12.3 [0.7–36.0]	13.7 ± 12.3 [1.7–37.1]	—
RRMS, *n* (%)	8 (57.1%)	8 (57.1%)	—
SPMS, *n* (%)	6 (42.9%)	6 (42.9%)	—
EDSS Score^b^	4.1 ± 2.1 [1–6.5]	3.7 ± 2.1 [0–6.5]	*p = *0.86
CES‐D	16.1 ± 13.0 [4–52]	13.1 ± 11.1 [4–40]	*p = *0.12

*Note*: Data are represented as mean ± standard deviation (SD) [range] unless otherwise indicated.

Abbreviations: CES‐D = Center for Epidemiologic Studies Depression; EDSS = Expanded Disability Status Scale; n* = *number of patients; RRMS = relapsing‐remitting multiple sclerosis; SPMS = secondary progressive multiple sclerosis.

^a^ Time from first symptom.

^b^ For EDSS ratings, there were 13 of the 14 subjects with a measurement at each time point, but only 12 had measurements at both time points. Therefore, the means and SDs are based on two different groups of 13, but the *p*‐value shown for the EDSS comparison is from *n = *12 using a paired *t*‐test. The EDSS comparison using a Wilcoxon signed‐rank test yielded a *p = *1.0.

**TABLE 2:** Previous disease modifying therapies taken before cladribine.

	Last DMT prior to cladribine start	Time on prior DMT (months)	Washout period (months)	Reason for change to cladribine
Patient 1	Interferon beta‐1a, rituximab, ocrelizumab^a^	59.99	5.62	Gradual symptom worsening
Patient 2	Ocrelizumab^a^	6.01	18.46	Unknown
Patient 3	Ocrelizumab^a^	12.02	6.54	Gradual symptom worsening
Patient 4	Ocrelizumab^a^	13.01	5.75	Logistics; patient left the country
Patient 5	Interferon beta‐1a, natalizumab, ocrelizumab^a^	32.00	7.82	Unknown
Patient 6	Glatiramer acetate, natalizumab, dimethyl fumarate, teriflunomide, ocrelizumab^a^	25.00	9.53	Unknown
Patient 7	Dimethyl fumarate^a^	14.13	29.21	Symptoms were not improving
Patient 8	Dimethyl fumarate^a^	10.64	2.33	Unknown
Patient 9	Interferon beta‐1a, glatiramer acetate, natalizumab, rituximab, alemtuzumab, dimethyl fumarate, ocrelizumab^a^	34.07	2.46	Symptoms were not improving
Patient 10	Natalizumab, teriflunomide, dimethyl fumarate, ocrelizumab^a^	34.56	5.55	Risk of PML, JCV antibody index high
Patient 11	Teriflunomide, fingolimod, teriflunomide, ocrelizumab^a^	18.04	14.00	Symptoms were not improving
Patient 12	Interferon beta‐1a, glatiramer acetate, fingolimod, ocrelizumab^a^	24.02	2.83	Symptoms were not improving
Patient 13	Interferon beta‐1a, natalizumab, teriflunomide^a^	77.01	9.82	Unknown
Patient 14	Ocrelizumab^a^	17.94	27.04	Self‐discontinued, did not want infusions

Abbreviations: DMT = disease modifying (immune) therapy for multiple sclerosis; JCV = John Cunningham virus; PML = Progressive multifocal leukoencephalopathy.

^a^ Last therapy before the start of cladribine.

All subjects completed the Center for Epidemiologic Studies Depression Scale (CES‐D) prior to cognitive testing. This is a 20‐question self‐report measure of depressive symptoms within the last week (Radloff 1977). The possible score range is 0–60, with a score of 16 or higher being considered in the depression range.

Subjects were administered the Brief Repeatable Battery of Neuropsychological Tests (BRB‐N) in MS and the Stroop Color and Word Test (Stroop). Tests were administered at baseline and ∼one year later. The time difference between the two visits was (mean ± standard deviation) [range] 1.0 ± 0.1 [0.7–1.3] years. Subjects started their first cladribine dose at an average of 1.5 ± 3.6 [−0.6 to 12.4] months before baseline cognitive testing and 14.0 ± 4.0 [11.6–26.4] months before follow‐up testing. Cognitive testing took place prior to the cladribine start date for ten subjects and after the cladribine start date for four subjects.

This study was approved by the Mass General Brigham Human Research Committee, and all participants gave written informed consent. Recruitment started on October 22, 2020, and was completed on July 17, 2023.

### 2.2 Brief Repeatable Battery of Neuropsychological Tests in MS

The BRB‐N includes the following five neuropsychological tests: Selective Reminding Test (SRT), 10/36 Spatial Recall Test (10/36), Symbol Digit Modalities Test (SDMT), PASAT, and Word List Generation (WLG) (Rao 1992). Stroop was also administered. (Golden 1976) When available (SRT, 10/36, SDMT, PASAT, and WLG), alternate form versions were used at each timepoint to reduce practice effects. Tests were administered by a research assistant (A.C. or S.K.) under the supervision of a PhD in Clinical Psychology (BIG).

The SRT is a verbal memory test. The subject is first read a list of 12 words by the examiner and is asked to recall as many of the words as they can. The subject is then read any words missed during that trial and again asked to recall all 12 words. There are six learning trials and a delayed recall trial. Scores include long‐term storage, consistent long‐term retrieval, and total delayed recall.

The 10/36 assesses visuospatial memory. A subject is shown a 6 × 6 square grid with 10 circles in specific locations for 10 seconds (s). They are then asked to recreate the design using 10 checker pieces on a blank square grid. There are three learning trials and a delayed recall trial. Scores are the total number correct on immediate recall and delayed recall.

The SDMT and PASAT assess information processing speed. During the oral version of the SDMT, subjects are provided with a key showing nine different symbols corresponding to the numbers 1–9; some symbols are mirror images of each other. Subjects are then given 90 s to substitute numbers for symbols according to the set code. Scores are the number of correct responses.

During the PASAT, the subject listens to a recording of 61 single‐digit numbers presented at a rate of one every 3 s. They are asked to add sequential pairs of numbers so that each digit is added to the digit that preceded it. For example, a subject will relay the sum of digits #1 and #2 out loud, followed by the sum of digits #2 and #3, then the sum of digits #3 and #4, and so on, until the recording ends. This is then repeated with the digits presented at a rate of one every 2 s. Scores are the total number of correct responses at the 3‐s rate and at the 2‐s rate.

The WLG task assesses verbal fluency. Subjects have one minute to produce as many words as possible, starting with a given letter of the alphabet. They cannot use proper names, numbers, or multiple forms of a word. There are three trials. Scores are the sum of correct words across the three trials.

### 2.3 Stroop Color and Word Test

Stroop examines both processing speed and executive function. For each part, subjects are presented with a piece of paper with 100 items split into 5 columns. Subjects have 45 s to read the columns of each page as fast as they can. During the first part, subjects are given a paper printed in black and white with only the words “red,” “blue,” and “green” in varied order. Subjects are asked to read the words on the page. During the second part, each word is replaced with 4 X's printed in colored ink (red, blue, or green). Subjects are asked to name the colors on the page. For the final part, the X's are replaced with a word printed in colored ink that does not match the word itself. For example, the word “red” might be printed in blue ink. Subjects are asked to name the color of the ink while ignoring the word itself. Scores are the number correct on each of the three parts.

### 2.4 Statistical Analysis

Paired *t*‐tests were used to compare baseline and follow‐up cognitive and EDSS data and the Wilcoxon signed rank test was used to further compare performance on EDSS between baseline and follow‐up. A *p*‐value of less than 0.05 was considered statistically significant.

## 3 Results

### 3.1 Neuropsychological Testing Results

On average, there was no worsening on any test after one year (Figure 1 and Table 3). Furthermore, both the PASAT 3‐second total correct (baseline 34.9 vs. follow‐up 38.3, *p = *0.07) and SRT total delayed recall (baseline 7.1 vs. follow‐up 8.2, *p = *0.05) showed a trend to improvement on‐study (Figure 2 and Table 3).

**FIGURE 1:**
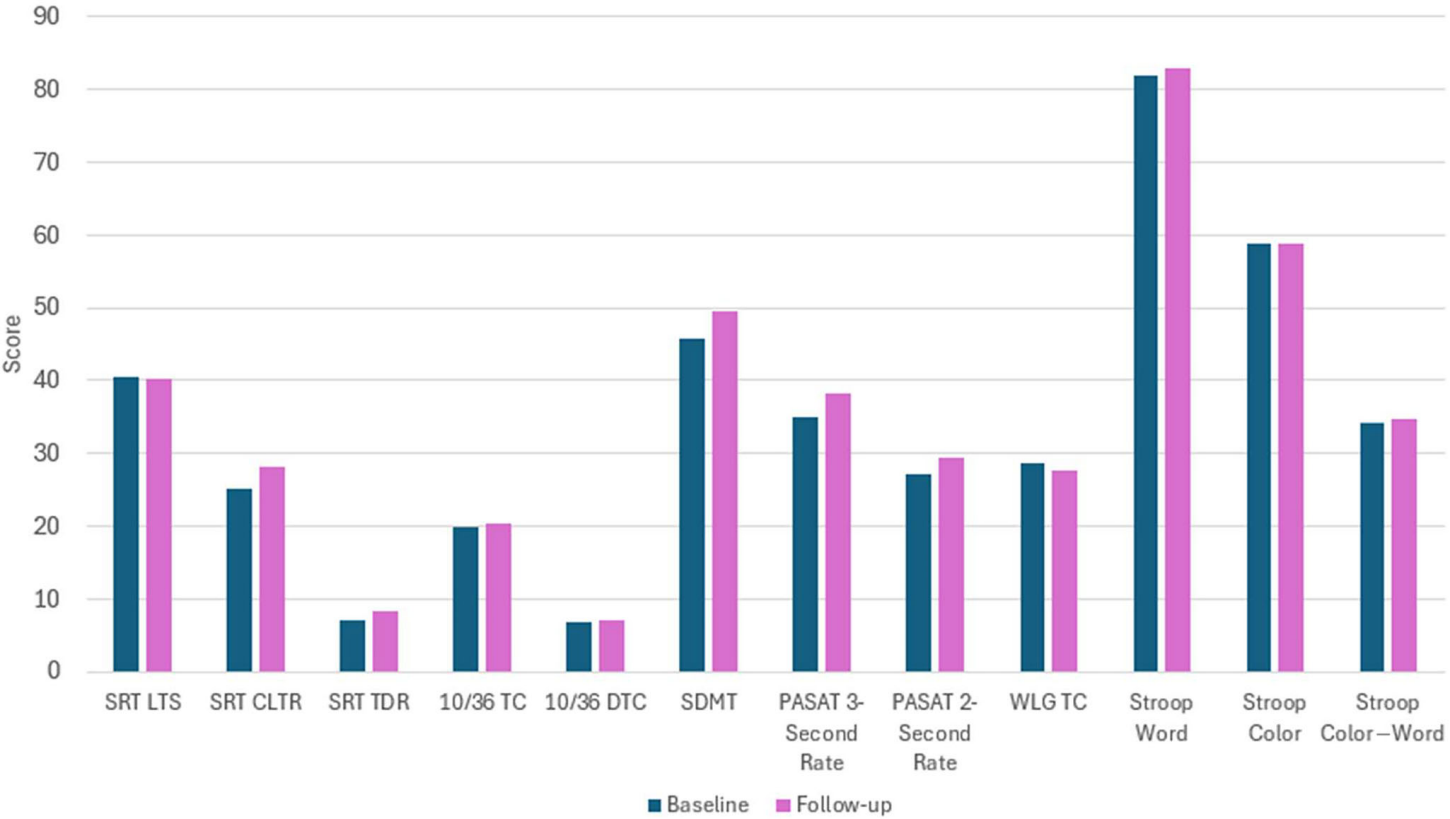
Cognitive testing measures over one year. Average cognitive scores over one year show no worsening from baseline to follow‐up. 2‐second rate = 2 s intervals between single‐digit numbers; 3‐second rate = 3 s intervals between single‐digit numbers; CLTR = consecutive long‐ term retrieval; DTC = delayed total correct; LTS = long‐ term storage; PASAT = Paced Auditory Serial Addition Test; SDMT = Symbol Digit Modalities Test; SRT = Selective Reminding Test; TC = total correct; TDR = total delayed recall; WLG = Word List Generation. See Table 3 for on‐study change (95% Confidence Interval) and *p*‐value for each measure.

**TABLE 3:** Cognitive measures.

	Baseline	Follow‐up	Follow‐up—Baseline change (95% CI); *p*‐value
SRT long‐term storage	40.6 ± 10.7	40.2 ± 14.6	−0.4 (−9.1, 8.4); *p = *0.93
SRT consecutive long‐term retrieval	25.1 ± 9.2	28.1 ± 12.5	3.0 (−2.8, 8.8); *p = *0.28
SRT total delayed recall	7.1 ± 1.9	8.2 ± 1.5	1.1 (−0.002, 2.1); *p = *0.05
10/36 total correct	19.9 ± 4.6	20.3 ± 5.2	0.4 (−2.6, 3.4); *p = *0.76
10/36 delayed total correct	6.7 ± 2.3	7.1 ± 4.0	0.4 (−1.4, 2.1); *p = *0.67
SDMT total correct	45.9 ± 12.3	49.6 ± 17.2	3.8 (−2.3, 9.9); *p = *0.20
PASAT 3‐second	34.9 ± 11.1	38.3 ± 9.1	3.4 (−0.3, 7.0); *p = *0.07
PASAT 2‐second	27.1 ± 5.9	29.4 ± 7.9	2.4 (−1.6, 6.3); *p = *0.22
WLG total correct	28.8 ± 8.8	27.6 ± 7.4	−1.1 (−4.9, 2.6); *p = *0.53
Stroop word total	82.0 ± 11.9	82.9 ± 13.6	0.9 (−4.0, 5.7); *p = *0.71
Stroop Color total	58.9 ± 12.8	58.8 ± 12.3	−0.1 (−7.0, 6.8); *p = *0.98
Stroop Color–Word total	34.2 ± 10.8	34.7 ± 9.9	0.5 (−2.4, 3.4); *p = *0.71

*Note*: Data are represented as mean ± standard deviation unless otherwise indicated. The confidence interval (CI) and *p*‐value are based on a paired t‐test.

Abbreviations: SRT = Selective Reminding Test; SDMT = Symbol Digit Modalities Test; PASAT = Paced Auditory Serial Addition Test; WLG = Word List Generation.

**FIGURE 2:**
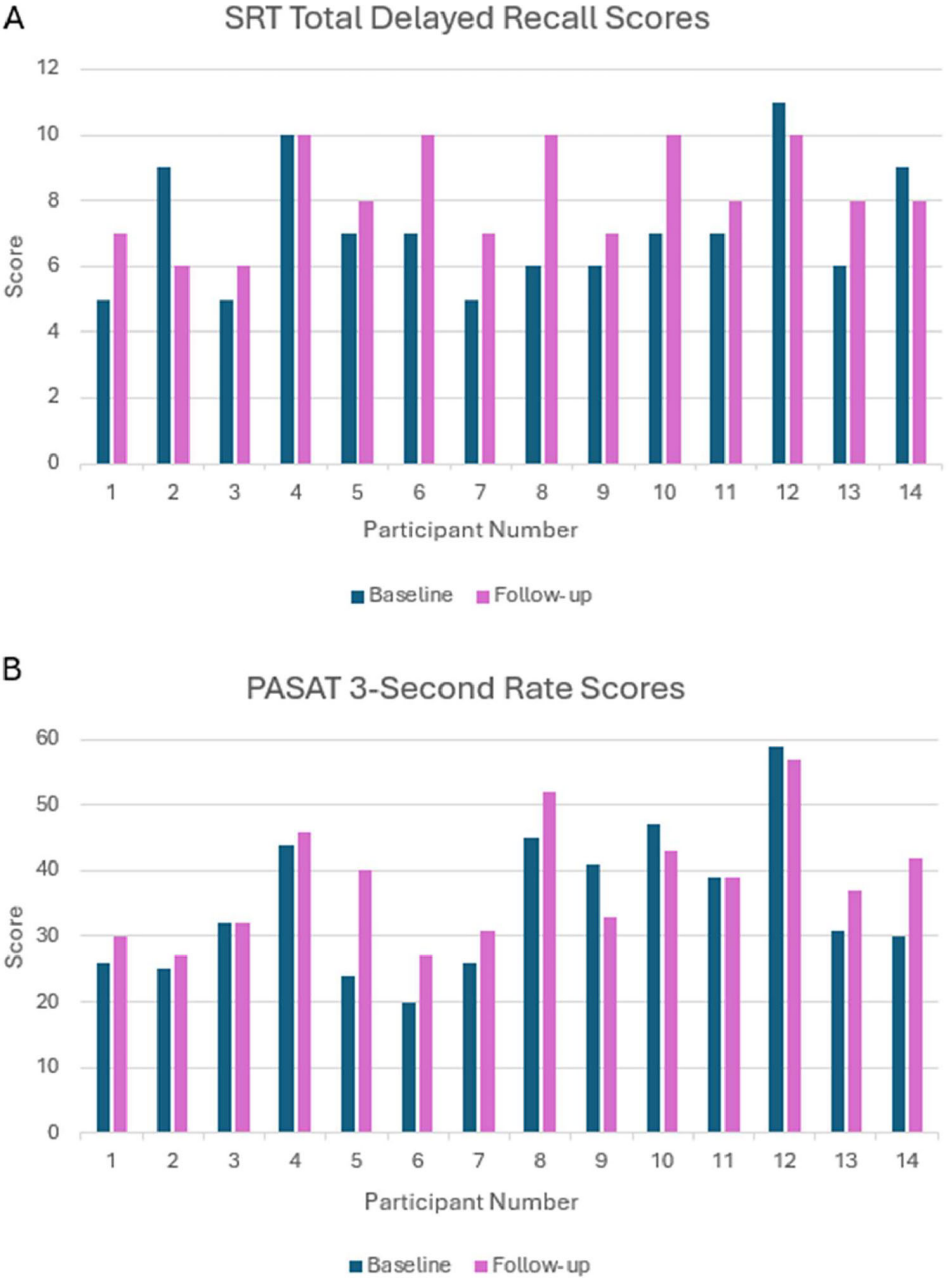
Baseline versus follow‐up scores for the Selective Reminding Test total delayed recall and Paced Auditory Serial Addition Test 3‐second rate. Individual participant scores at both baseline and follow‐up are shown for Selective Reminding Test (SRT) total delayed recall (A) and Paced Auditory Serial Addition Test 3‐second intervals between single‐digit numbers (PASAT 3‐second rate) (B). Both SRT total delayed recall and PASAT 3‐second rate trended towards score increases on‐study (SRT total delayed recall: 1.1 score increase *p = *0.05; PASAT 3‐second rate: 3.4 score increase *p = *0.07). Each x‐axis number represents an individual participant.

### 3.2 Neurologic Disability and Relapses

EDSS score did not differ significantly after one year (Table 1). No patients had clinical relapses on‐study.

### 3.3 Depression Scores

Scores on the CES‐D did not differ significantly after one year (baseline 16.1 ± 13.0 vs. follow‐up 13.1 ± 11.1; *p = *0.12, Table 1). A total of 28.6% (*n = *4) of patients scored greater than 16 on the CES‐D for at least one timepoint. Of the four participants, three scored above 16 at both baseline and follow‐up visits. One participant scored above 16 at baseline, but below at follow‐up, and one participant scored below 16 at baseline, but greater than 16 at follow‐up.

## 4 Discussion

Our study suggests preservation of cognitive function over one year in patients with MS newly started on cladribine tablets. At baseline, this cohort of patients had fairly advanced disease, encompassing participants with both relapsing and progressive forms of MS, a moderate level of physical disability and long disease duration, on average.

During the observation period, there was no worsening on any of the cognitive measures and a trend towards higher PASAT 3‐seconds and SRT delayed recall task scores. This is in line with a previous study that found that processing speed test *z*‐scores were stable over time in cladribine‐treated patients tested every six months over two years (Conway et al. 2025).

A potential explanation for such stability may relate to an impact of cladribine therapy on preserving whole brain GM volume, including deep GM volume, and limiting new white matter and cortical lesions, each of which is implicated in cognitive performance and is thought to be impacted by cladribine (Freedman et al. 2021; Comi et al. 2018; Zurawski et al. 2025; Barrantes‐Cepas et al. 2025). Cortical GM atrophy, hippocampal and deep GM nuclei atrophy are associated with cognitive impairment (Eijlers et al. 2018; Damjanovic et al. 2017), with thalamic atrophy being most closely associated with processing speed, working memory, and visuospatial memory (Houtchens et al. 2007). Thus, cladribine's treatment effect on volume loss and inflammation could contribute to cognitive preservation over time and help explain the stability seen in this short‐term study.

Importantly, studies to date on the impact of cladribine tablets on cognition have assessed their cohorts over periods of less than 2.5 years. Our study had a short‐term observation of cognitive change (1 year); thus, it is important to question whether cognitive decline is typically seen in this time period in MS patients. The study by Podda et al. (2024) identified different cognitive phenotypes and found that cognitive performance across phenotypes did not decline very much over one year, even though some declines in performance were noted. That study, however, did not consider DMT usage, which could have impacted these results. Amato et al. (2001) assessed cognitive impairment over 10 years in a mixed MS cohort of treated or untreated patients and found that the percentage of impaired patients increased from 26% at baseline to 49% after 4 years, to 56% ten years after baseline. Cognitive impairment usually progresses over time, but the potential decline over one year is not clear. While this is likely an adequate observation period to assess MRI effects, longer term studies may be necessary to fully understand the effect on cognition such as limiting the rate of decline or promoting improvement.

Furthermore, while preserving GM volume could prevent future cognitive impairment, it might not restore lost cognitive functioning independently. This could help explain stability in cognitive scores in this cohort, but not marked improvement, over one year. Restorative therapies such as cognitive rehabilitation in combination with cladribine may be of interest to examine in future studies (De Meo et al. 2025).

Numerous studies have assessed the impact of DMTs on cognitive function in MS. The Landmeyer et al. (2020) meta‐analysis included longitudinal studies to compare lower vs. higher efficacy therapies (not including newer drugs such as cladribine and ocrelizumab) on cognitive performance in relapsing–remitting MS, mostly assessing information processing speed. Both treatment groups showed improved cognitive performance from baseline to follow‐up, without any between‐group difference.

In a study of early‐stage patients with MS, Glasmacher et al. (2022) assessed the impact of DMT on “hidden” disability burden, defined as depression, pain, anxiety, fatigue, sleep difficulties, and cognitive changes. Participants were grouped into three hidden disability classes: low, moderate, and high. The majority of participants were unchanged in their level of hidden disability burden (70.2%), with about one‐fourth showing improvement (26.1%). When using a 3‐category DMT classification system based on purported efficacy, treatment with cladribine tablets or fingolimod (moderate efficacy; Category 2) was associated with improvement in hidden disability class level. These results may indirectly support our findings. However, given the methodologic differences between the two studies, no definitive conclusions can be reached.

There are several notable limitations to our study. This was a single‐center, single‐arm, real‐world study with a small sample size. There was no control group to make direct comparisons, such as to other MS DMT groups or healthy individuals. Furthermore, practice effects make interpretation of findings difficult, especially with the PASAT (Bever et al. 1995), because it is known that taking the PASAT a second time improves performance (Baird et al. 2007). However, to mitigate this practice effect, tests were administered one year apart and with different versions of tests, when available. Furthermore, statistically significant changes also do not necessarily translate into clinically meaningful improvements. For the SDMT, early studies suggested a 4‐point change, while a more recent paper suggests using a higher threshold that accounts for individual differences (Strober et al. 2022). In addition, due to the real‐world logistics of executing the study, one patient had a potentially confounding baseline for cognitive testing, which was performed well after the start of cladribine. Finally, participants had complex treatment histories before the start of cladribine (e.g., ocrelizumab, natalizumab, alemtuzumab). Therefore, it is possible that there may have been some carryover effects on cognition from previous immunotherapies prior to making a transition to cladribine. Taken together, these limitations suggest that the results may not be completely representative of standard cladribine initiation and should be considered exploratory and preliminary. Future studies are needed to determine the extent to which changes in cognitive test performance relate to real‐world outcomes such as quality of life and employment status. Longitudinal studies with a larger cohort are needed to fully elucidate the effects of cladribine tablets on cognitive performance.

## 5 Conclusion

In this small exploratory cohort, no clear decline in cognitive performance was detectable over one year in patients newly started on cladribine tablets.

## Author Contributions

R.B. conceived the study and secured the funding. B.G. performed study supervision and performed cognitive testing. B.C.H. provided statistical analysis. A.C. and S.K. performed cognitive testing. R.B., A.C., and B.G. drafted the manuscript. A.C., B.G., and S.T. collected data. A.C. and S.T. consented subjects. A.J. assisted in collecting clinical data. R.B., S.T., F.M., S.N., S.R., T.S., and J.Z. assisted with patient recruitment. R.B. and J.Z. performed the clinical exams. All authors contributed to data interpretation, reviewed and approved the final version of the manuscript.

## Funding

This study was funded by EMD Serono (ROR identifier: https://ror.org/027zrs220).

## Ethics Statement

This study was approved by the Mass General Brigham Human Research Committee, and all participants gave written informed consent.

## Consent

This study was approved by the Mass General Brigham Human Research Committee (Protocol 2009P001530), and all participants gave written informed consent.

## Conflicts of Interest

Dr. Healy has received research support from Novartis and Sanofi/Genzyme. Dr. Houtchens has received consulting honoraria from Genentech, EMD Serono, Novartis, and TG therapeutics, and research support from Biogen, Novartis, and Roche/Genentech. Dr. Mateen has received research funding from Bristol‐Myers Squibb, EMD Serono, Genentech, Horizon Therapeutics, Novartis, and TG Therapeutics, and has consulted for Alexion, EMD Serono, Genentech, and Horizon Therapeutics. Dr. Napoli has received speaking honoraria from EMD Serono, Biogen, Eisai, TG Therapeutics, and Bristol Myers Squibb. Dr. Rizvi has received advisory board consulting fees from Amgen and TG Therapeutics. Dr. Singhal has received research support from Novartis, EMD Serono, and Genzyme‐Sanofi, payment for consulting from Novartis, Genentech, EMD Serono, Genzyme‐Sanofi, TG Therapeutics, Eisai, and Biogen, and speaking fees from Tiziana Life Sciences. Dr. Zurawski has received research support from Novartis and I‐Mab Biopharma. Dr. Bakshi has received speaking honoraria from EMD Serono, advisory board consulting fees from Sanofi, and research support from Novartis. The remaining authors declare no conflicts of interest.

## Data Availability

The data that support the findings of this study will be considered to be made available upon request to the corresponding author. The data are not publicly available.
